# Occupational endotoxin exposure in association with atopic sensitization and respiratory health in adults: Results of a 5-year follow-up

**DOI:** 10.1371/journal.pone.0189097

**Published:** 2017-12-06

**Authors:** Elisabeth A. J. Spierenburg, Lidwien A. M. Smit, Esmeralda J. M. Krop, Dick Heederik, Machteld N. Hylkema, Inge M. Wouters

**Affiliations:** 1 Institute for Risk Assessment Sciences, Division of Environmental Epidemiology, Utrecht University, Utrecht, the Netherlands; 2 Department of Pathology, University Medical Center Groningen, University of Groningen, Groningen, the Netherlands; 3 GRIAC- Groningen Research Institute for Asthma and COPD, University Medical Center Groningen, University of Groningen, Groningen, the Netherlands; Universite de Bretagne Occidentale, FRANCE

## Abstract

The objective of the present longitudinal study was to investigate the effects of occupational endotoxin exposure on respiratory health and atopic sensitization in adults. Health outcomes and personal endotoxin exposure estimates were determined for 234 farmers and agricultural workers both at baseline and 5 years later. A questionnaire was used to assess respiratory symptoms, spirometry tests were performed and total and specific IgE levels were measured in serum.

A twofold increase in personal endotoxin exposure was associated with less hay fever (OR 0.68, 95%CI 0.54-0.87) and grass IgE positivity (OR 0.81, 95%CI 0.68-0.97) at both time points (“persistent” versus “never”). Although not statistically significant, a consistent protective pattern was observed for an increased loss of hay fever symptoms (OR 2.19, 95%CI 0.96-4.99) and grass IgE positivity (OR 1.24, 95%CI 0.76-2.02), and for less new-onset of hay fever (OR 0.87, 95%CI 0.65-1.17), grass IgE positivity (OR 0.83, 95%CI 0.61-1.12) and atopic sensitization (OR 0.75, 95%CI 0.55-1.02). Endotoxin exposure was not associated with changes in lung function.

We showed that occupational endotoxin exposure is associated with a long-term protective effect on hay fever and grass IgE positivity. Results on longitudinal changes in hay fever, atopy and grass IgE positivity in adulthood were consistent with a protective effect of endotoxin exposure, but results need to be confirmed in larger cohorts. An effect of endotoxin exposure on lung function decline was not found.

## Introduction

Numerous studies in children and adults have shown a protective effect of farm related exposures on atopy and allergic symptoms.[1–3] Furthermore, an ongoing protective effect of farm childhood on allergic symptoms is also observed later in life.[4,5] Similar protective effects have also been observed in studies among adults with high occupational exposure, independent of farm childhood.[4–6] The farm environment is characterized by a high exposure to micro-organisms. Exposure to endotoxin, a cell wall component of Gram negative bacteria, has been shown to explain the ‘farming effect’ to some extent, both in children and adults.[2,4,5] However, these studies were mainly cross-sectional and only few longitudinal studies assessed effects of farming exposure on allergic responses over time. One longitudinal study in young adults found a protective effect of farming exposure on new-onset sensitization to any of four inhalant allergens.[6] Another longitudinal study in children showed a protective effect of parental farming on prevalence and new-onset skin prick test (SPT) positivity for any of six inhalant allergens and some evidence of increased loss of SPT positivity in children living on a farm.[1] However, these studies allow conclusions on (parental) farming in general, but do not allow evaluation of specific factors and exposure levels in relation to the observed health effects.

Besides being protective for allergic responses, occupational farming related exposures are known to have adverse effects on respiratory health as well,[7–12] a phenomenon that was investigated in a few longitudinal studies as well. And although previous studies showed an accelerated decline in FEV_1_ and FVC in farmers compared to non-farmers, exposure-response relations were mostly not included.[9–12] Only a few studies measured endotoxin exposure and the results of these studies are inconclusive. Some showed that annual decline of FEV_1_, FVC and FEF_25-75_ were significantly associated with endotoxin levels,[7,8] whereas another observed no effect.[13] A recent systematic review and meta-analysis also showed that conclusions regarding exposure-response relationships for organic dust exposure and lung function changes could not be drawn. Yet, for organic dust exposed individuals versus controls a significant but small effect on FEV_1_, but not on other lung function parameters, was shown.[14]

We established a cohort consisting of farmers and agricultural workers in 2005. In the baseline cross-sectional analysis of this cohort we found an increased risk of prevalent wheeze, but a decreased prevalence of hay fever and atopic sensitization. In particular grass pollen sensitization had a decreased prevalence in relation to endotoxin exposure.[4,5] The objective of the current longitudinal study is to investigate the relationship between measured occupational endotoxin exposure and changes over 5 years in atopic sensitization, lung function and respiratory symptom status.

## Materials and methods

### Study design

A five-year follow-up study was conducted in endotoxin exposed workers. At both time points participants filled in the same questionnaire [15] and underwent a health exam consisting of a lung function measurement and collection of a blood sample to determine serum IgE levels. Endotoxin exposure was measured in a subset of participants at both time points.

The study protocol was approved by the University Medical Centre Utrecht ethics committee and all participants gave written informed consent both at baseline and follow-up.

### Study population

#### Baseline

The study population consisted of an occupational cohort of Dutch Caucasian farmers and agricultural industry workers recruited in 2005-2006 as described previously.[5] Shortly, farmers were either livestock farmers raising cattle, pigs, chickens or goats, and/or crop farmers growing fruit, vegetables or grain. The agricultural companies were involved in flower bulb, onion, animal feed or seed processing.

At baseline 453 participants (95 farmers and 358 agricultural workers) took part in the study with a questionnaire and health exam.[4]

#### Follow-up

Follow-up was conducted in 2010-2011. Individual farmers and companies participating at baseline were approached again.[15] 80 out of 95 farmers (84%) and 19 out of 23 companies (83%) agreed to participate. At these 19 participating companies 109 (38%) participants had left their job and were not available for follow-up, the remaining 62% did participate. We previously compared baseline health characteristics between participants and those that were unavailable for follow-up which showed no major differences between the two groups, indicating there is no healthy worker selection effect in our cohort.[15]

To be included in the study, participants needed to at least have completed the questionnaire and either lung function, serological data or both had to be available. This resulted in 234 participants: 75 farmers and 159 agricultural workers. The number of persons included in each analysis varies depending on the participation in the different subparts of the study. We did not differentiate between farmers and company workers.

### Questionnaire

At both baseline and follow-up, the questionnaire was sent by mail prior to the health exam.[5] It contained questions on general characteristics, farm childhood, allergies, asthma, smoking habit and respiratory symptoms. Questions on respiratory symptoms were adopted from the Dutch version of the ECRHS questionnaire.[16] Hay fever was defined as self-reported pollen allergy accompanied by itchy or watery eyes or sneezing. Wheeze was established by a positive answer to the question “Did you experience wheeze in the past twelve months?” Allergy was determined by a positive answer to the question “Have you ever had any allergies?” For the complete questionnaire see S1 and S2 Texts.

### Serum IgE

Total IgE and specific IgE against house dust mite (HDM), grass pollen (grass), cat, and dog antigens were analyzed in serum by ELISA.[17] Specific serum IgE exceeding an optical density of 0.1 were classified as positive. Atopic sensitization or atopy was defined as positive specific serum IgE for any of the tested allergens. To avoid bias due to methodological and batch differences, we re-analyzed the baseline sera together with the follow-up sera.

### Lung function

Lung function measurements were performed in the morning. Forced spirometry was performed at baseline and follow-up using the same pneumotachograph and software (Jaeger, Würzburg, Germany). All tests were evaluated according to European Respiratory Society standards.[18] Lung function curves were considered acceptable if back extrapolated volume was small (<5% FVC and <150 ml) and expiratory time was at least 4 seconds.[19] A lung function measurement was considered reproducible if ΔFEV_1_ and ΔFVC between curves were <150ml and ΔPEF was either <670 ml or 10% of maximum measured PEF. Only acceptable and reproducible lung function measurements were used for the analyses. In total, 231 participants performed a lung function measurement both at baseline and follow-up of which 161 (69.7%) were acceptable using the above criteria at both occasions. A sensitivity analysis showed that selection based only on ΔPEF <670 ml or only including curves with at least 6 seconds expiratory time did not change the results (data not shown).

The Global Lungs Initiative reference values (GLI, 2012) were used to determine percentage of predicted values for FEV_1_, FVC, FEV_1_/FVC and FEF_25-75_ (forced expiratory flow during 25-75% of FVC), e.g. FEV_1_%pred.[20] The cut-offs for FEV_1_ and FVC below the lower limit of normal (<LLN) were set at <5% of the GLI population.

### Endotoxin exposure assessment

#### Exposure measurement

Full-shift personal inhalable dust samples were collected at baseline (n = 249) and at follow-up (n = 127) in a subset of participants (see S1 Table). As described previously,[5] Gilian GilAir portable pumps (Gilian, West Caldwell, NJ, USA) were used with Gesamt Staub Probenehmer (GSP) sampling heads and 37-mm glass-fibre filters (Whatman GF/A, Maidstone, UK). Filters were extracted in pyrogen-free water with 0.05% Tween-20.[21] Supernatants were analyzed by the quantitative kinetic chromogenic Limulus amebocyte lysate assay without applying Tween in the assay solution.[21]

#### Exposure modelling

A job-exposure matrix was constructed using a linear mixed regression model. No major changes took place in the process lines or exposure control equipment (ventilation) in the companies or farms between baseline and follow-up. Therefore, we modeled exposure based on measurements from both time points as these were regarded as repeated measurements of the same jobs.[22] Modeled endotoxin exposure values per job title are presented in S1 Table.

Each participant was assigned an exposure level both at baseline and at follow-up based on job title.

Relationships between endotoxin exposure and changes in respiratory morbidity or sensitization status were investigated using baseline exposure as the main exposure during the follow-up period. Twenty-five percent of the participating workers changed exposure during the follow-up period (26 participants (11%) changed to a job with higher exposure and 32 participants (14%) changed to a job with lower exposure). Therefore, additional analyses were performed with inclusion of change in exposure (Δexposure) during the follow-up period as well. This method allowed us to correct for both ‘baseline level of exposure’ and a change in exposure over time separately, both of which may be linked to a change in outcome. Change in exposure (Δexposure) was calculated by subtracting endotoxin exposure levels at baseline from the levels at follow-up.

### Data analysis

Changes in general characteristics of the study population between baseline and follow-up were investigated using a paired student t-test for continuous variables and McNemar’s Chi-squared test for paired data for categorical variables.

Continuous outcomes (change per year in lung function parameters and total IgE) were analyzed by linear regression. Dichotomous outcomes (questionnaire results and specific IgE positivity) were analyzed by logistic regression. For this, outcomes were summarized in four categories: never (absent at baseline and follow-up), new-onset (negative at baseline and positive at follow-up), loss (positive at baseline and negative at follow-up) and persistent (positive both at baseline and follow-up). We were interested in the effect of exposure on a possible change in outcome over time, and on persistence of outcomes. Therefore participants who had changed during follow-up (new-onset or loss) were compared to participants with the same baseline condition who did not change over time, being respectively never and persistent for new-onset and loss. In addition, we estimated the effect of endotoxin exposure on persistent outcomes compared to those who never had the outcome. Analyses were adjusted for age, gender, smoking and farm childhood. Wheeze was additionally adjusted for atopy. Change or persistence in the dichotomous confounders smoking and atopy were summarized in the same way as the dichotomous outcomes.

Endotoxin exposure and total IgE levels in serum were log-normally distributed and therefore log base 2 transformed before analysis. Thus odds ratios have been calculated for a two-fold increase in exposure.

Data was analyzed using SAS 9.4.

## Results

### General characteristics

Characteristics of the study population at baseline and follow-up are shown in Table 1. The mean follow-up time was 4.8 years. Six smokers at baseline had quit smoking at follow-up. Five participants reported farm childhood at baseline, but did not report this at follow-up. For the analysis, baseline farm childhood was used. A small, but not significant, decrease in endotoxin exposure of 39 EU.m^-3^ (p = 0.19) between baseline and follow-up was observed. A significant decline in total IgE level was found between baseline and follow-up. The population prevalence of specific sensitization, atopy, self-reported allergy and hay fever remained the same.

**Table 1:** General characteristics of the study population at baseline and follow-up. Differences between baseline and follow-up are tested for statistical differences: paired t-test for continuous variables, McNemar’s paired chi squared test for binary outcomes. The number of persons included in each analysis depends on the source of the outcome data: questionnaire (n = 234), lung function (n = 161), or serological data (n = 212).

		Baseline (2005–2006)		Follow-up (2010–2011)		
	N					p
Age (years; mean, SD)	234	41.9	10.0	46.7	10.1	
Gender (female; n, %)	234	28	12.0%	28	12.0%	
BMI (kg.m^-2^; mean, SD)	161	26.2	3.6	27.0	3.7	**<0.01**
Current smoker (n, %)	234	59	25.2%	53	22.6%	0.06
Farm childhood (n, %)	234	133	56.8%	128	54.7%	0.17
Endotoxin exposure (EU.m^-3^; GM, GSD)	234	314.9	4.8	276.0	5.1	0.19
FEV_1_						
L (mean, SD)	161	4.1	0.9	3.9	0.8	**<0.01**
% pred (GLI; mean, SD)	161	98.8	14.4	97.4	14.7	**0.03**
<LLN (GLI; n, %)	161	14	8.7%	16	9.9%	0.74
FVC						
L (mean, SD)	161	5.4	1.1	5.2	1.1	**<0.01**
% pred (GLI; mean, SD)	161	102.9	12.6	101.7	13.8	0.09
<LLN (GLI; n, %)	161	3	1.9%	6	3.7%	0.37
PEF						
L.s^-1^ (mean, SD)	161	10.8	2.2	10.4	2.2	**<0.01**
% pred (mean, SD)	161	118.6	19.2	115.6	18.5	**0.01**
FEV_1_/FVC						
x100 (mean, SD)	161	77.0	6.7	76.1	8.0	**<0.01**
% pred (GLI; mean, SD)	161	95.6	8.3	95.5	9.9	0.86
<LLN (GLI; n, %)	161	19	11.8%	20	12.4%	0.56
FEF_25-75_						
L.s^-1^ (mean, SD)	161	3.5	1.4	3.5	1.3	0.94
% pred (GLI; mean, SD)	161	86.4	30.5	92.6	31.5	**<0.01**
<LLN (GLI; n, %)	161	21	13.0%	16	9.9%	0.05
Self-reported allergy (n, %)	234	65	27.8%	68	29.1%	0.56
Hay fever (n, %)	234	28	12.0%	28	12.0%	1.00
Atopy (n, %)	212	66	31.1%	67	31.6%	0.82
Total IgE (IU/ml; GM, GSD)	212	25.1	7.4	23.3	6.4	**0.02**
HDM IgE positive (n, %)	212	47	22.2%	43	20.3%	0.21
Grass IgE positive (n, %)	212	41	19.3%	41	19.3%	1.00
Cat IgE positive (n, %)	212	4	1.9%	6	2.8%	0.16
Dog IgE positive (n, %)	212	2	0.9%	3	1.4%	0.32

SD: standard deviation

GM: geometric mean

GSD: geometric standard deviation

LLN: lower limit of normal

Lung function generally decreased in the years between baseline and follow-up (all p<0.01 except for FEF_25-75_ p = 0.94, Table 1). The decline in FEV_1_ was larger than that of the GLI reference population, which is reflected in the decline of FEV_1_%pred (p<0.05).This was observed to a lesser extent for FVC%pred (p = 0.09). There were no significant changes in the number of lung function values <LLN. There was a slight decrease in reported current asthma (p = 0.02), and an increase in reporting of wheeze (p = 0.03).

### Changes in reported health symptoms and serum IgE between baseline and follow-up

The number of participants in each outcome category is shown in Table 2. For all outcomes, the majority of participants were negative at baseline and follow-up. The number of participants with new onset or loss of symptoms or positive serum IgE was small (0-8%). The prevalence of persistent atopy, HDM IgE and grass IgE was 27%, 19% and 15%, respectively. Self-reported allergy was reported at both occasions by 22% of participants.

**Table 2:** Changes in reported health symptoms and IgE positivity between baseline and follow-up. Participants are categorized as never (absent at baseline and follow-up), new onset (negative at baseline and positive at follow up), loss (positive at baseline and negative at follow up) and persistent (positive both at baseline and follow up) health symptoms.

	N	Never	New onset	Loss	Persistent
		n (%)	n (%)	n (%)	n (%)
Asthma (past 12 months)	234	210 (90%)	4 (2%)	12 (5%)	8 (3%)
Wheeze (past 12 months)	212	168 (79%)	18 (8%)	7 (3%)	19 (9%)
Hay fever	234	197 (84%)	9 (4%)	9 (4%)	19 (8%)
Self-reported allergy	234	153 (65%)	16 (7%)	13 (6%)	52 (22%)
Atopy	212	136 (64%)	10 (5%)	9 (4%)	57 (27%)
HDM IgE	212	162 (76%)	3 (1%)	7 (3%)	40 (19%)
Grass IgE	212	162 (76%)	9 (4%)	9 (4%)	32 (15%)
Cat IgE	212	206 (97%)	2 (1%)	0 (0%)	4 (2%)
Dog IgE	212	209 (99%)	1 (0%)	0 (0%)	2 (1%)

### Consistent protective pattern for hay fever, atopy and grass IgE

**Fig 1:**
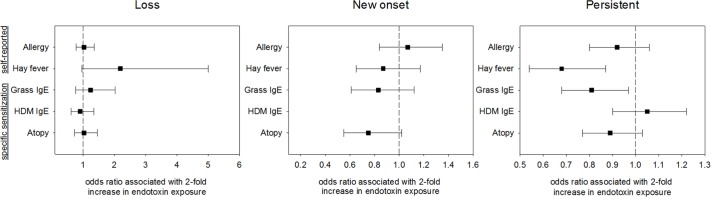
Odds of loss, new onset and persistence of allergic outcomes during follow-up in association with endotoxin exposure. Analyses are adjusted for potential confounders (age, gender, smoking and farm childhood). A consistent protective pattern is observed for hay fever, grass IgE sensitization and atopy, although not all associations meet statistical significance.

The associations between endotoxin exposure and hay fever, grass IgE sensitization and atopy indicate a consistent protective pattern (Table 3, allergic symptoms graphically represented in Fig 1), although not all associations met statistical significance. Higher endotoxin exposure is associated with loss of hay fever symptoms (OR 2.19, p = 0.06). Moreover, higher endotoxin exposure results in fewer individuals with persistent hay fever (OR 0.68, p = 0.002), and seems to result in less new-onset of hay fever symptoms over time (OR 0.87, p = 0.36). These protective effects on hay fever symptoms were independent of changes in exposure during the follow-up period. A similar protective pattern was observed for specific sensitization to grass pollen (loss: OR 1.24 p = 0.39, persistent: OR 0.81 p = 0.02, new-onset: OR 0.83 p = 0.23), showing consistency between self-reported hay fever and grass sensitization, an objective measure of hay fever. A protective pattern was also observed for persistent and new onset of atopic sensitization (loss: OR 1.03 p = 0.86, persistent: OR 0.89 p = 0.11, new-onset: OR 0.75 p = 0.07). No consistent pattern was observed for self-reported allergy, asthma, wheeze or HDM IgE. Decline in total IgE was also not associated with endotoxin exposure (Table 4).

**Table 3:** Association of endotoxin exposure and reported health symptoms and specific IgE positivity. Adjusted logistic regression analysis; OR’s are associated with a 2-fold increase in endotoxin exposure. Analyses are adjusted for potential confounders (age, gender, smoking and farm childhood). Wheeze is additionally adjusted for atopy.

	New-onset vs never				Loss vs persistent				Persistent vs never			
	Baseline exposure		Baseline exposure adjusted for Δexposure		Baseline exposure		Baseline exposure adjusted for Δexposure		Baseline exposure		Baseline exposure adjusted for Δexposure	
	OR	95% CI	OR	95% CI	OR	95% CI	OR	95% CI	OR	95% CI	OR	95% CI
**Self-reported symptoms**												
Asthma	n < 5 data not shown				0.74	(0.46–1.20)	0.82	(0.47–1.43)	1.11	(0.83–1.49)	1.11	(0.83–1.49)
Wheeze	0.88	(0.70–1.11)	0.87	(0.69–1.09)	0.74	(0.42–1.29)	*0*.*22*	*(0*.*04–1*.*22)*	1.13	(0.91–1.40)	1.13	(0.91–1.40)
Allergy	1.07	(0.84–1.35)	1.08	(0.85–1.37)	1.03	(0.78–1.35)	0.99	(0.74–1.32)	0.92	(0.80–1.06)	0.93	(0.81–1.07)
Hay fever	0.87	(0.65–1.17)	0.87	(0.65–1.17)	*2*.*19*	*(0*.*96–4*.*99)*	1.85	(0.81–4.22)	**0.68**	**(0.54–0.87)**	**0.69**	**(0.54–0.87)**
**Sensitization**												
Grass IgE	0.83	(0.61–1.12)	0.82	(0.60–1.12)	1.24	(0.76–2.02)	1.18	(0.72–1.94)	**0.81**	**(0.68–0.97)**	**0.82**	**(0.69–0.98)**
HDM IgE	n < 5 data not shown				0.91	(0.62–1.34)	0.90	(0.61–1.34)	1.05	(0.90–1.22)	1.06	(0.91–1.24)
Atopy*	*0*.*75*	*(0*.*55–1*.*02)*	*0*.*72*	*(0*.*52–1*.*00)*	1.03	(0.73–1.46)	1.02	(0.71–1.45)	0.89	(0.77–1.03)	0.90	(0.77–1.04)

Italic type: p<0.1; Bold type: p<0.05.

*Positive for any of the four allergens cat, dog, grass or HDM.

**Table 4:** Association of change in lung function or total IgE with endotoxin exposure. Adjusted linear regression analysis: betas represent changes associated with a 2-fold increase in endotoxin exposure. Analyses are adjusted for possible confounders (age, gender, smoking and farm childhood).

		Baseline exposure		Baseline exposure adjusted for Δexposure	
	N	Beta	p	beta	p
**Δ FEV**_**1**_					
mL.year^-1^	161	3.35	0.20	2.87	0.28
% pred (GLI).year^-1^	161	0.02	0.75	0.01	0.83
**Δ FVC**					
mL.year^-1^	161	0.45	0.90	-0.54	0.88
% pred (GLI).year^-1^	161	-0.05	0.51	-0.06	0.38
**ΔFEV**_**1**_**/FVC**					
.year^-1^	161	0.05·10^−3^	*0*.*07*	0.06·10^−3^	*0*.*05*
% pred (GLI).year^-1^	161	0.07	*0*.*06*	0.08	**0.04**
**ΔFEF**_**25-75**_					
.year^-1^	161	0.01	**0.01**	0.01	**0.01**
% pred (GLI).year^-1^	161	0.27	**0.04**	0.27	**0.04**
**Δ Total IgE**					
IU.year^-1^	212	-0.01	0.87	0.03	0.68

### Lung function

Only modest effects of endotoxin exposure on changes in lung function were observed (Table 4). Apart from the general observation that FEV_1_ declines slightly more rapidly in our study population than in the GLI reference population, no association between ΔFEV_1_ and endotoxin exposure was observed. We also did not observe an association between endotoxin exposure and ΔFVC.

We found that an increase in baseline exposure tended to be associated with a very small increase in ΔFEV_1_/FVC (β = 0.5·10^−3^) and ΔFEV_1_/FVC%pred (β = 0.07). Inclusion of Δexposure did not change these estimates. An increase in baseline exposure was also associated with a small increase in ΔFEF_25-75_ and ΔFEF_25-75_%pred over time (β = 0.01 and β = 0.27 respectively), irrespective of inclusion of Δexposure in the model.

## Discussion

This is one of the first studies to investigate longitudinal changes in atopic sensitization and allergic responses in association with measured personal endotoxin exposure levels in an occupationally exposed adult population. Our results show evidence of a protective effect of endotoxin exposure on new onset and persistent atopic sensitization and hay fever independent of farm childhood. We found no major effects of endotoxin exposure on lung function decline.

One of the main advantages of our study is the longitudinal design, as most studies to date have examined relationships between endotoxin exposure and allergic diseases in a cross-sectional set-up.

Another strength of our study is that the blood samples from baseline were analyzed together with the samples from follow-up avoiding methodological differences in assay performance. This is also an advantage over skin prick tests, which are used in most studies. Skin prick tests are necessarily conducted and measured at the separate time points, allowing for the introduction of measuring differences based on batch differences in extracts and differences between test conductors.[23] Compared to skin prick testing, IgE ELISA testing of all samples at the same time is more consistent.

An advantage of this study compared to other longitudinal studies is that we collected exposure measurements and job title information at both baseline and follow-up. As discussed by Bolund *et al*. in their meta-analysis, even longitudinal studies generally do not include a change in exposure in their analyses.[14] Job title information at both time points enabled us to estimate exposure levels at both time points as well as changes in exposure between baseline and follow-up (in case of a change in job title). It can be hypothesized that the new-onset or loss of symptoms or specific serum IgE can be partially attributed to a change in exposure level. Our results do not indicate a major impact of exposure changes on investigated health outcomes, as effect estimates of baseline exposure changed only marginally with inclusion of Δexposure in the models (Table 3), and effect estimates of Δexposure were small (see S2 and S3 Tables). Results should be interpreted cautiously due to limited study size and because timing of job changes within the 5 year follow-up period is unknown. Yet outcomes indicate that in the current study exposure misclassification due to job changes were relative small or had little impact, this may imply that effects are not likely promptly. Whether this is typical for the current study or a general phenomenon is hard to identify as most longitudinal studies do not take changes of exposure into account during data analysis.[14]

One of the limitations of our study is the relatively modest size of our study population. Additionally, in the majority of participants, health outcomes were stable between baseline and follow-up, resulting in a small number of participants in the categories of change (new-onset and loss), limiting the power of these analyses. We therefore did not adjust for multiple testing and did not focus on the effects of exposure change However, the fact that we found a consistent pattern for hay fever, atopy and grass IgE positivity is in line with our expectations of a protective effect of endotoxin exposure on allergy.

The power to pick up changes in lung function is also limited by the study size and duration of follow-up. Surprisingly, we observed a small positive relationship between ΔFEV_1_/FVC and endotoxin exposure which is reflected in an improvement in ΔFEV_1_/FVC%pred. Additionally, we found a positive but very small effect of endotoxin exposure on ΔFEF_25-75_, which is also reflected in ΔFEF_25-75_%pred. In contrast to our study, some previous longitudinal studies reported an accelerated decline in ΔFEV_1_/FVC [24,25]or an accelerated decline in FEF_25-75_ [7,26] in relation to endotoxin exposure. Given that the regression coefficients in our study are very small and the analyses are only just significant, we do not believe these to be biologically relevant. We did not find an effect of endotoxin exposure on FEV_1_ or FVC decline. Some previous longitudinal studies observed an accelerated decline in FEV_1_ in relation to endotoxin exposure either when using exposure classification [10, 12, 24, 26] or measured endotoxin exposure,[7,8] whereas other longitudinal studies using categorical exposure found no effect of farming on FEV_1_ decline.[9,11,13] The studies that did find an accelerated effect of farming on FEV_1_ observed small effects ranging from an additional -0.71 ml/yr FEV_1_ for animal feed handling within farmers [24] to an additional -26.1 ml/yr FEV_1_ for swine confinement workers versus non farming controls.[26] The longitudinal studies which used measured exposure found an effect of -0.326 ml/yr FEV_1_ per mg.m^3^ endotoxin exposure in grain and animal feed industry [7] and -19 ml/yr per factor 2 increase of endotoxin exposure [8] respectively. The absence of major long-term effects of organic dust exposure on lung function is confirmed by a recent meta-analysis by Bolund *et al*. which showed only a small significant effect of organic dust exposure on FEV_1_ of 4.92 ml/yr for exposed versus controls.[14] However, results were inconsistent for quantitative exposure measures and accelerated lung function decline. The effect of farming or endotoxin exposure on lung function remains inconclusive.

Healthy worker selection could be of influence on the health effects observed in this study, possibly leading to an underestimation of effect estimates. Earlier analyses on this cohort, which focused on selective loss to follow-up did not reveal major health related differences between participants included in follow-up and those lost to follow-up, indicating that there is no major healthy worker survival going on in our population during the time of our study.[15] Also no significant differences or trends were found in baseline demographic characteristics, respiratory health or allergies between participants changing to jobs with lower exposure and those moving to jobs with similar or higher exposure.[15] Nonetheless, pre-study selection such as healthy worker hire selection cannot be excluded and we have no information on potential changes in health of those lost to follow-up. However, both our previous cross-sectional analyses [4,5] and our loss to follow-up analysis [15] do not give us reason to suspect a large pre-study selection effect.

Whether adult exposure to farm dust or endotoxin has a protective effect is still under debate.[27] Several cross-sectional studies in adults have linked farming exposures to a reduced likelihood of developing asthma, atopy and allergies, but the results are inconsistent and could possibly be explained by healthy worker survival effects or lingering effects from childhood exposures.[27–29] Yet, the epidemiological evidence for a protective effect in adults is growing.[5,6,30–32]

In our previously published cross-sectional analyses of the same cohort, we found that higher endotoxin exposure protects against hay fever and atopic sensitization.[4,5] In the present longitudinal analysis, these protective effects were confirmed. Although not all relationships were statistically significant, they consistently indicated a protective effect of endotoxin exposure on new-onset and persistence of hay fever, atopy and grass IgE positivity. Moreover, we also showed a tendency for increased loss of IgE positivity in association with higher endotoxin exposures during the follow-up period. Only a few other longitudinal studies investigated allergic responses in farm environments, albeit none of those included exposure-response relationships. In a small follow-up study in 42 farmers,[30] IgE-mediated sensitization was observed less frequently at follow-up and no new sensitizations occurred. Similar to our and most other studies on the “farming effect”,[27] this was most pronounced for grass sensitization. No such effect was observed for HDM sensitization which is consistent with other studies.[27] A longitudinal study in farmers [6] also found a protective effect of exposure on new-onset sensitization. However, this study classified exposure as being a current farmer or ex-farmer compared with non-farming control subjects. In children, a protective effect of parental farming on prevalence of atopy and new-onset skin prick test positivity was shown as well as some evidence of increased loss of positivity in children living on a farm.[1] In our current longitudinal study, we show that adult endotoxin exposure contributes to protection against development or persistence of atopy and allergies and that this effect is independent of healthy worker effects or farm childhood (see S4 and S5 Tables). Although we have identified relationships with endotoxin, other exposures, like different types of farm dust, could correlate with endotoxin and therefore be responsible for the observed effects of endotoxin exposure. However, two recent studies showed an association between asthma and lower microbial diversity in nasal swab and mattress dust,[28,29] where a higher microbial diversity was encountered in a farming environment. This seems to indicate that microbial exposures, such as endotoxin, play an important role in allergic desensitization.

The mechanisms through which endotoxin or farming exposure protect against allergic outcomes is not yet elucidated. It has been suggested that the protective effect of endotoxin exposure on allergic diseases is mediated through balancing Th1 and Th2 responses in the immune system in early life.[33] As this effect is most pronounced and part of the natural development of the infant immune system, it was long thought unlikely that endotoxin exposure in adulthood could also be protective against allergic diseases.

In addition to regulation of the Th1/Th2 balance during childhood, the epidemiological evidence for a protective effect in adults is growing.[5,6,30–32] Additionally, studies investigating the mechanism behind the protective effect of endotoxin exposure on allergic diseases in adults have found a shifted Th1/Th2 balance in farmers compared to non-farming controls consistent with a lower prevalence of allergic diseases in farmers and agricultural workers.[34] Thus, the regulation of the immune response might be strongest in childhood, but could continue throughout life.

In conclusion, in this occupational cohort of agricultural workers and farmers, we showed a longitudinal protective effect of endotoxin exposure on hay fever, atopy and grass IgE positivity. Our longitudinal study provides further evidence that adult endotoxin exposure may protect against the development of and persistent absence of allergy in adulthood, which should however be reconfirmed in a larger cohort.

## Supporting information

S1 Text Questionnaire on work and health for farmers and agricultural industry workers, original Dutch version. (PDF)

S2 Text Questionnaire on work and health for farmers and agricultural industry workers, English translation. (PDF)

S1 Table Exposure estimates using baseline model and pooled model by industry and job title. (PDF)

S2 Table Association of baseline endotoxin exposure and Δexposure with reported health symptoms and specific IgE positivity. (PDF)

S3 Table Linear regression analysis of lung function and total IgE in association with baseline endotoxin exposure and Δexposure. (PDF)

S4 Table Adjusted logistic regression analysis of reported health symptoms and specific IgE positivity in association with endotoxin exposure with and without additional adjustment for farm childhood. (PDF)

S5 Table Linear regression analysis of lung function and total IgE in association with endotoxin exposure with and without additional adjustment for farm childhood. (PDF)

S1 Dataset (XLSX)
