# Supplementary material for: Occupational endotoxin exposure in association with atopic sensitization and respiratory health in adults: Results of a 5-year follow-up
Source: PLoS One. 2017 Dec 6;12(12):e0189097. doi: 10.1371/journal.pone.0189097 (PMC5718503; doi:10.1371/journal.pone.0189097)
Supplement: S1 Text — (PDF) [file pone.0189097.s001.pdf]

Vragenlijst over werk en gezondheid voor agrariërs en medewerkers in de agrarische industrie

In deze vragenlijst staan vragen over uw persoonsgegevens en uw werkzaamheden (onderdeel A, B en C), vragen over luchtwegklachten en allergieën (onderdeel D en E), en over uw rookgewoonten (onderdeel F).
De delen A, D, E en F zijn voor alle deelnemers gelijk, de delen B en C verschillen per sector.

Als er nog onduidelijkheden zijn, dan kunt u altijd even bellen of een e-mail sturen.

Institute for Risk Assessment Sciences
Universiteit Utrecht

Universiteit Utrecht

Wanneer u deze vragenlijst niet wilt invullen, dan vragen wij u de - niet-ingevulde - vragenlijst terug te sturen, met de reden waarom u niet wilt meewerken. Op deze manier weten wij of alle lijsten zijn aangekomen en kunnen we inschatten of de redenen van mensen om wel of niet mee te werken, een vertekend beeld kunnen geven van het uiteindelijke onderzoeksresultaat.

- o Ik wil deze vragenlijst niet invullen, omdat..……….

  ....

Alle deelnemers

A) ALGEMENE VRAGEN

A1) Wat is uw geslacht?

- o Man
- o Vrouw

A2) Wat is uw geboortedatum? _____ _____ 19______ (dag, maand, jaar)

A3) Bent u opgegroeid op een boerderij/agrarisch bedrijf?

- o Ja → Ga door naar vraag A3 a
- o Nee → Ga door naar vraag B1

A3 a) Wat was de voornaamste productie activiteit op het bedrijf waar u bent opgegroeid? *(Meerdere antwoorden mogelijk)*

- o Veehouderij
- o Plantaardige productie

Boeren

B) VRAGEN OVER UW BEDRIJF

(Onderdeel B hoeft alleen ingevuld te worden door één van de eigenaren van het bedrijf)

B1) Hoeveel mensen werken er op dit bedrijf, inclusief uzelf? ………… (aantal)

B2) Heeft u in het huidige bedrijf eerst op gangbare wijze geproduceerd, en is het bedrijf later omgeschakeld naar biologische productie?

- o Ja → Ga door naar vraag B2a
- o Nee → Ga door naar vraag B3

B2a) Hoeveel jaar geleden bent u begonnen met het omschakelen naar biologische productie? ………. (jaar geleden)

B3) Wat is of wat zijn de voornaamste productie-activiteit(en) van uw huidige bedrijf? Gebeurt deze productie op biologische, biologisch-dynamische, gedeeltelijk biologische, of gangbare wijze? *(Meerdere antwoorden mogelijk)*

Soort productie	Biologisch	Biologisch-dynamisch	Gedeeltelijk biologisch	Gangbaar
o Melkveehouderij	o	o	o	o
o Rundveehouderij, vleesvee	o	o	o	o
o Pluimveehouderij, legkippen	o	o	o	o
o Pluimveehouderij, vleeskuikens	o	o	o	o
o Fokzeugenhouderij	o	o	o	o
o Vleesvarkenshouderij	o	o	o	o
o Geiten of schapenhouderij, melkvee	o	o	o	o
o Geiten of schapenhouderij, vleesvee	o	o	o	o
o Akkerbouw: granen	o	o	o	o
o Akkerbouw: veevoedergewassen	o	o	o	o
o Akkerbouw: overige	o	o	o	o
o Vollegrondsgroenteteelt	o	o	o	o
o Bloembollenteelt	o	o	o	o
o Boomteelt	o	o	o	o
o Fruitteelt	o	o	o	o
o Glastuinbouw	o	o	o	o
o Paddestoelenteelt	o	o	o	o
o Anders, nl.	o	o	o	o

B4) Hoeveel van de volgende landbouwhuisdieren houdt u op uw bedrijf?

Soort landbouwhuisdier	Aantal
Koeien	....
Stieren	....
Kalveren en pinken	....
Legkippen	....
Vleeskuikens	....
Ander pluimvee, nl...................................	....
Fokzeugen	....
Mestvarkens	....
Geiten	....
Schapen	....
Paarden	....
Andere dieren, nl...................................	....
Andere dieren, nl...................................	....

B5) Maakt u gebruik van desinfecterende middelen in uw bedrijf?

- ○ Ja → Ga door naar vraag B5 a
- ○ Nee → Ga door naar vraag C1

B5 a) Welke desinfecterende middelen gebruikt u?
Kruis alleen de middelen aan die u regelmatig gebruikt.
(Meerdere antwoorden mogelijk)

○	QAC (Deosan, P3-Incidin)
○	QAC + logen (Incosept, Nofordes)
○	QAC + aldehyden (Macrodes, Megades, Favoriet Ontsmetal, Parvotech, Aldekol, CID 20, Neoseptal, Roloxid, Togodor)
○	Chloramine-T (Halamid)
○	Chloorverbindingen (Actisan, Sumatox, Chloortabletten, natriumhypochloriet)
○	Loog+chloorverbindingen (Quick Clean, Neomosan)
○	Overige desinfecterende middelen, nl ……………………………………………………………………..

Boeren

C) VRAGEN OVER UW WERK

C1) Hoe oud was u toen u begon met werken op een agrarisch bedrijf? ……….. (jaar)

C2) Hoe oud was u toen u begon met werken op het bedrijf waar u nu werkt? ……….. (jaar)

C3) Bent u (mede)eigenaar van het bedrijf waar u nu werkt?
- o Ja
- o Nee

C4) Hoeveel uur per week werkt u op het bedrijf waar u nu werkt?
- o Minder dan 20 uur per week
- o 20 tot en met 32 uur per week
- o 33 tot en met 40 uur per week
- o Meer dan 40 uur per week

C5) Welke van de volgende handelingen **voert u zelf uit**?
(Meerdere antwoorden mogelijk)

Soort handeling	
o Dierverzorging	o Werken met vloeibare mest
o Werken met houtspaanders of zaagsel	o Werken met droge mest
o Werken met stro	o Werken met bestrijdingsmiddelen
o Werken met hooi	o Gewasverzorging, vollegrond
o Werken met kuilgras	o Gewasverzorging, kassen
o Zelf mengen van diervoeder	o Werkzaamheden met graan
o Ruimtes desinfecteren	o Uien of bloembollen sorteren
o Schoonmaken met hoge druk spuit	o Aardappelen sorteren
o Schoonmaken met brandslang	o Andere werkzaamheden op een akkerbouwbedrijf
o Geen van deze handelingen, mijn hoofdtaak in het bedrijf is: ……………………………………..………………………………	

C6) Wat is volgens u het meest stoffige werk dat u uitvoert?

……………………………………..……………………….………………………..…………………

C7) Maakt u regelmatig gebruik van persoonlijke beschermingsmiddelen tijdens uw werk?
(Meerdere antwoorden mogelijk)
- o Ja, handschoenen
- o Ja, adembescherming
- o Nee

Bloembollen sector

B) VRAGEN OVER UW WERK

B1) Wanneer bent u bij het huidige bedrijf begonnen met werken?

_____ _____ ______ (dag, maand, jaar)

B2) Hoeveel uur per week werkt u?
- o Minder dan 20 uur per week
- o 20 tot en met 32 uur per week
- o 33 tot en met 40 uur per week
- o Meer dan 40 uur per week

B3) Wat is uw huidige functie?

o	Heftruckchauffeur
o	Productie medewerker
o	Verpakkingsmedewerker
o	Technische dienst, onderhoudsmonteur
o	Huishoudelijke dienst
o	Teelt-/Oogstmedewerker
o	Laboratorium medewerker
o	Administratief medewerker
o	Leidinggevende (bedrijfsleider, directeur)
o	Anders, nl:……………………………

B4) Welke van de volgende werkzaamheden voert u uit, en hoe vaak?

	Nooit	Maximaal een halve dag per week	1 tot 3 dagen per week	4 tot 5 dagen per week
○ Gewasverzorging, teelt, oogst	○	○	○	○
○ Heftruck besturen	○	○	○	○
○ Bloembollen spoelen	○	○	○	○
○ Bloembollen sorteren	○	○	○	○
○ Verpakken, handmatig	○	○	○	○
○ Verpakken, machinaal	○	○	○	○
○ Reparatie, onderhoud	○	○	○	○
○ Schoonmaken: opruimen/vegen	○	○	○	○
○ Schoonmaken: hoge druk spuit	○	○	○	○
○ Schoonmaken: perslucht	○	○	○	○
○ Schoonmaken: brandslang	○	○	○	○
○ Laboratorium werkzaamheden	○	○	○	○
○ Kantoorwerk	○	○	○	○
○ Anders, nl.	○	○	○	○

B5) Waar in het bedrijf werkt u het merendeel van de tijd (<u>één antwoord aankruisen</u>)?

○ Buiten
○ Ontvangsthal
○ Productieafdeling
○ Verpakkingsafdeling
○ Verzendhal/magazijn
○ Werkplaats
○ Laboratorium
○ Kantoor
○ Ergens anders, nl:……………………………..

Diervoeder

B) VRAGEN OVER UW WERK

B1) Wanneer bent u bij het huidige bedrijf begonnen met werken?

_____ _____ ______ (dag, maand, jaar)

B2) Hoeveel uur per week werkt u?

- o Minder dan 20 uur per week
- o 20 tot en met 32 uur per week
- o 33 tot en met 40 uur per week
- o Meer dan 40 uur per week

B3) Wat is uw huidige functie?

o Vrachtwagenchauffeur
o Kraanmachinist
o Losser schip (bobcat)
o Productie medewerker / procesoperator
o Opzakker
o Heftruckchauffeur
o Technische dienst, onderhoudsmonteur
o Corveeër / siloschoonmaker
o Laboratorium- / kwaliteitsmedewerker
o Administratief medewerker
o Leidinggevende (bedrijfsleider, directeur)

B4) Welke van de volgende werkzaamheden voert u uit, en hoe vaak?

	Nooit	Maximaal een halve dag per week	1 tot 3 dagen per week	4 tot 6 dagen per week
○ Grondstoffen lossen	○	○	○	○
○ Controle kamer	○	○	○	○
○ Bijstorten	○	○	○	○
○ Heftruck besturen	○	○	○	○
○ Opzakken/afzakken	○	○	○	○
○ Vrachtauto's beladen	○	○	○	○
○ Reparatie, onderhoud	○	○	○	○
○ Schoonmaken: opruimen/vegen	○	○	○	○
○ Schoonmaken: stofzuigen	○	○	○	○
○ Schoonmaken: perslucht (kleding)	○	○	○	○
○ Laboratorium werkzaamheden	○	○	○	○
○ Kantoorwerk	○	○	○	○
○ Machines perserij, losserij, mengerij, afzakken	○	○	○	○

B5) Waar in het bedrijf werkt u het merendeel van de tijd (<u>één antwoord aankruisen</u>)?

○ Buiten (lossen schepen)
○ Laden / lossen bulkwagens
○ Controlekamer
○ Fabriekshal
○ Opzak / afzak
○ Verzendhal
○ Werkplaats
○ Laboratorium
○ Kantoor

Uiensector
B) VRAGEN OVER UW WERK

B1) Wanneer bent u bij het huidige bedrijf begonnen met werken?

_____ _____ ______ (dag, maand, jaar)

B2) Hoeveel uur per week werkt u?

- o Minder dan 20 uur per week
- o 20 tot en met 32 uur per week
- o 33 tot en met 40 uur per week
- o Meer dan 40 uur per week

B3) Wat is uw huidige functie?

o Heftruckchauffeur
o Productie medewerker
o Verpakkingsmedewerker
o Technische dienst, onderhoudsmonteur
o Huishoudelijke dienst
o Teelt-/Oogstmedewerker
o Laboratorium medewerker
o Administratief medewerker
o Leidinggevende (bedrijfsleider, directeur)
o Anders, nl:……………………………

B4) Welke van de volgende werkzaamheden voert u uit, en hoe vaak?

	Nooit	Maximaal een halve dag per week	1 tot 3 dagen per week	4 tot 5 dagen per week
○ Gewasverzorging, teelt, oogst	○	○	○	○
○ Heftruck besturen	○	○	○	○
○ Uien afstaarten	○	○	○	○
○ Uien sorteren	○	○	○	○
○ Verpakken	○	○	○	○
○ Reparatie, onderhoud	○	○	○	○
○ Schoonmaken: opruimen/vegen	○	○	○	○
○ Schoonmaken: hoge druk spuit	○	○	○	○
○ Schoonmaken: perslucht	○	○	○	○
○ Schoonmaken: brandslang	○	○	○	○
○ Laboratorium werkzaamheden	○	○	○	○
○ Kantoorwerk	○	○	○	○
○ Anders, nl.	○	○	○	○

B5) Waar in het bedrijf werkt u het merendeel van de tijd (<u>één antwoord aankruisen</u>)?

○ Buiten
○ Ontvangsthal
○ Productieafdeling
○ Verpakkingsafdeling
○ Verzendhal/magazijn
○ Werkplaats
○ Laboratorium
○ Kantoor
○ Ergens anders, nl:……………………………..

Zaaizaadsector

B) VRAGEN OVER UW WERK

B1) Wanneer bent u bij het huidige bedrijf begonnen met werken?

_____ _____ ______ (dag, maand, jaar)

B2) Hoeveel uur per week werkt u?

- o Minder dan 20 uur per week
- o 20 tot en met 32 uur per week
- o 33 tot en met 40 uur per week
- o Meer dan 40 uur per week

B3) Wat is uw huidige functie?

o Heftruckchauffeur
o Productie medewerker/ proces operator
o Verpakkingsmedewerker
o Technische dienst, onderhoudsmonteur
o Huishoudelijke dienst
o Gewasverzorger
o Laboratorium medewerker
o Administratief medewerker
o Leidinggevende (bedrijfsleider, directeur)
o Anders, nl:……………………………

B4) Welke van de volgende werkzaamheden voert u uit, en hoe vaak?

	Nooit	Maximaal een halve dag per week	1 tot 3 dagen per week	4 tot 5 dagen per week
○ Gewasverzorging, teelt, oogst	○	○	○	○
○ Heftruck besturen	○	○	○	○
○ Kantelen, storten	○	○	○	○
○ Schonen	○	○	○	○
○ Mengen	○	○	○	○
○ Zaden ontsmetten, coaten	○	○	○	○
○ Verpakken	○	○	○	○
○ Reparatie, onderhoud	○	○	○	○
○ Schoonmaken: opruimen/vegen	○	○	○	○
○ Schoonmaken: stofzuigen	○	○	○	○
○ Schoonmaken: perslucht	○	○	○	○
○ Laboratorium werkzaamheden	○	○	○	○
○ Kantoorwerk	○	○	○	○
○ Anders, nl..	○	○	○	○

B5) Waar in het bedrijf werkt u het merendeel van de tijd (<u>één antwoord aankruisen</u>)?

○ Buiten
○ Kas
○ Ontvangsthal
○ Productieafdeling, proceshal
○ Verpakkingsafdeling
○ Verzendhal/magazijn
○ Werkplaats
○ Laboratorium
○ Kantoor
○ Ergens anders, nl:……………………………..

Alle sectoren

B6) Wat is volgens u het meest stoffige werk dat u uitvoert?

………………………………………..…………………….………………….…………………

B7) Maakt u regelmatig gebruik van persoonlijke beschermingsmiddelen tijdens uw werk? *(*Meerdere antwoorden mogelijk*)*

- o Ja, handschoenen
- o Ja, adembescherming
- o Nee

B8) Heeft u voordat u deze baan kreeg in een ander bedrijf in de bloembollen sector gewerkt?

- o Ja → Hoe lang? …………Jaar
- o Nee

B9) Heeft u voordat u in de bloembollen sector ging werken nog een andere baan gehad waarbij u in aanraking kwam met stof?
Bijvoorbeeld bakkerij, graanindustrie, landbouw, etc.

- o Ja → Ga door naar vraag B9 a
- o Nee

B9 a) Wat voor bedrijf of bedrijven?

…………………………………………..	Hoelang?	………….Jaar
…………………………………………..	Hoelang?	………….Jaar
…………………………………………..	Hoelang?	………….Jaar

Alle sectoren

C) VRAGEN OVER PERSOONLIJKE HYGIENE

C1) Eet of drinkt u op de werkplek (dus buiten het kantoor of de kantine)?
- o Ja, altijd
- o Ja, soms
- o Nee

C2) Doucht u meestal aan het eind van de werkdag?
- o Ja, op het werk
- o Ja, thuis
- o Nee

C3) Wordt uw werkkleding door het bedrijf of thuis gewassen?
- o Door het bedrijf
- o Deels door het bedrijf, deels thuis
- o Thuis

C4) Kleedt u zich thuis of op het werk om?
- o Op het werk
- o Thuis

D) VRAGEN OVER GEZONDHEIDSKLACHTEN

D1) Heeft u wel eens problemen met de ademhaling?

- o Voortdurend, zodat uw ademhaling nooit helemaal in orde is
- o Herhaaldelijk, maar het gaat altijd weer helemaal over
- o Nooit / Slechts zelden

D2) Heeft u de afgelopen 12 maanden wel drie maanden achtereen vrijwel dagelijks gehoest?

- o Ja
- o Nee

D3) Bent u in de afgelopen 12 maanden wel eens wakker geworden door een hoestbui?

- o Ja
- o Nee

D4) Heeft u de afgelopen 12 maanden wel drie maanden achtereen vrijwel dagelijks slijm opgehoest?

- o Ja
- o Nee

D5) Bent u de afgelopen maanden wel eens wakker geworden met een gevoel van beklemming op de borst?

- o Ja
- o Nee

D6) Heeft u last van kortademigheid wanneer u zich op vlak terrein moet haasten, of wanneer u een lichte helling of een trap in normale pas oploopt?

- o Ja → Ga door naar vraag D6 a
- o Nee → Ga door naar vraag D7

D6a) Heeft u wel eens last van kortademigheid wanneer u met leeftijdsgenoten in normaal tempo op vlak terrein wandelt?

- o Ja
- o Nee

D7) Heeft u gedurende het afgelopen jaar wel eens last gehad van piepen op de borst?

- o Ja → Ga door naar vraag D7 a
- o Nee → Ga door naar vraag D8

D7 a) Was u kortademig tijdens dit piepen op de borst?

- o Ja
- o Nee

D7 b) Heeft u dit piepen op de borst gehad terwijl u niet verkouden was?

- o Ja
- o Nee

D8) Heeft u ooit van werk moeten veranderen als gevolg van bronchitis of emfyseem?

- Ja
- Nee

D9) Heeft u in de afgelopen 12 maanden geneesmiddelen voor bronchitis of emfyseem gebruikt?

- Ja → Ga door naar vraag D9 a
- Nee → Ga door naar vraag D10

D9 a) Welke geneesmiddelen heeft u gebruikt voor bronchitis of emfyseem?
Meerdere antwoorden mogelijk

- Ventolin
- Atrovent
- Bricanyl
- Prednison
- Anders, nl..

D10) Bent u in het afgelopen jaar wel eens wakker geworden door een aanval van kortademigheid?

- Ja
- Nee

D11) Heeft u in het afgelopen jaar wel eens overdag in rust een aanval van kortademigheid gehad?

- Ja
- Nee

D12) Heeft u in het afgelopen jaar wel eens na inspanning een aanval van kortademigheid gehad?

- Ja
- Nee

D13) Heeft u ooit astma gehad?

- Ja → Ga door naar vraag D13 a
- Nee → Ga door naar vraag D14

D13 a) Werd de astma door een arts bevestigd?

- Ja → Ga door naar vraag D13 b
- Nee → Ga door naar vraag D13 c

D13 b) Hoe oud was u toen de diagnose astma werd gesteld? …………….jaar

D13 c) Heeft u in de afgelopen 12 maanden een astma-aanval gehad?

- Ja → Ga door naar vraag D13 d
- Nee → Ga door naar vraag D13 f

D13 d) Hoeveel astma aanvallen heeft u in de afgelopen 12 maanden gehad? …………….

D13 e) Heeft u de afgelopen 12 maanden tijdens uw werk wel eens een astma aanval gehad?

- ○ Ja
- ○ Nee

D13 f) Gebruikt u momenteel geneesmiddelen (bijv. inhalatoren, aerosols, tabletten) voor astma?

- ○ Ja → Ga door naar vraag D13 g
- ○ Nee → Ga door naar vraag D14

D13 g) Welke geneesmiddelen heeft u gebruikt voor astma?
Meerdere antwoorden mogelijk

- ○ Ventolin
- ○ Pulmicort
- ○ Atrovent
- ○ Bricanyl
- ○ Becotide
- ○ Symbicort
- ○ Flixotide
- ○ Prednison
- ○ Anders, nl...

D14) Bent u overgevoelig of allergisch (geweest) voor één of meer stoffen?

- ○ Ja → Ga door naar vraag D14 a
- ○ Nee → Ga door naar vraag D15

D14 a) Zo ja, wilt u dan aankruisen welke stoffen dat zijn en aangeven wat de aard van de klachten is die u daarbij heeft?
Meerdere antwoorden mogelijk.

	Niezen of loopneus	Benauwdheid (astma)	Jeukende of rode huid	Jeukende of tranende ogen
○ Huisstof	○	○	○	○
○ Voedsel	○	○	○	○
○ Dieren	○	○	○	○
○ Planten of graspollen	○	○	○	○

D14 b) Bent u de afgelopen 12 maanden bij een huisarts of specialist geweest vanwege allergische klachten?

- ○ Ja
- ○ Nee

D14 c) Heeft u de afgelopen 12 maanden wel eens uw werk moeten verzuimen vanwege allergische klachten?

- ○ Ja
- ○ Nee

D15) Heeft u ooit van werk moeten veranderen als gevolg van astma?

- Ja
- Nee

D16) Heeft u ooit van werk moeten veranderen als gevolg van allergie?
- Ja
- Nee

D17) Heeft u ooit van werk moeten veranderen als gevolg van andere gezondheidsklachten?
- Ja
- Nee

D18) Heeft u in de afgelopen 12 maanden last (gehad) van piepen op de borst, kortademigheid of beklemd gevoel van de borst als gevolg van bepaalde werkzaamheden of werkplekken?
- Ja → Ga door naar vraag D18 a
- Nee → Ga door naar vraag D19

D18 a) Met welke werkplekken of werkzaamheden brengt u deze klachten in verband?

…………………………………………………………………………………

D18 b) Wanneer zijn de symptomen begonnen?
- Binnen 4 uur na aanvang van het werk
- Meer dan 4 uur na aanvang van het werk

D19) Heeft u gedurende de afgelopen 12 maanden last gehad van plotselinge aanvallen van griepachtige verschijnselen, zoals koortsaanvallen, rillingen, malaise, spier- of gewrichtspijn, <u>waarbij u zich weer geheel beter voelde binnen 1-2 dagen?</u>
- Ja → Ga door naar vraag D19 a
- Nee → Ga door naar vraag D20

D19 a) Met welke werkplekken of werkzaamheden brengt u deze klachten in verband?

…………………………………………………………………………………

D19 b) Hoe vaak is dit in de afgelopen 12 maanden voorgekomen? …………… keer

D 20) Hoe vaak heeft u tijdens de afgelopen 12 maanden last gehad van een of meer van de volgende symptomen?
EN
Verergeren deze klachten tijdens of na uw werk?

	Hoe vaak heeft u last van deze klacht?				Verergert het tijdens of na uw werk?	
	Dagelijks of bijna elke dag	1 tot 2 keer per week	1 tot 2 keer per maand	Nooit / zelden	Nee	Ja
1. Droge hoest	O	O	O	O	O	O
2. Slijm ophoesten	O	O	O	O	O	O
3. Piepen op de borst	O	O	O	O	O	O
4. Kortademigheid met piepen	O	O	O	O	O	O
5. Kortademigheid	O	O	O	O	O	O
6. Benauwd	O	O	O	O	O	O
7. Verstopte neus	O	O	O	O	O	O
8. Loopneus	O	O	O	O	O	O
9. Prikkend gevoel in neus of niezen	O	O	O	O	O	O
10. Droge keel of keelpijn	O	O	O	O	O	O
11. Jeukende, prikkende, tranende ogen	O	O	O	O	O	O
12. Jeukende of rode huid meer dan 2 dagen	O	O	O	O	O	O
13. Huiduitslag	O	O	O	O	O	O
14. Hoofdpijn	O	O	O	O	O	O
15. Zwaar/drukkend gevoel in hoofd	O	O	O	O	O	O
16. Duizeligheid	O	O	O	O	O	O
17. Ongewoon gevoel van moeheid	O	O	O	O	O	O
18. Concentratiemoeilijkheden	O	O	O	O	O	O
19. Koorts	O	O	O	O	O	O
20. Rillerig, aanvallen van rillingen	O	O	O	O	O	O
21. Pijn in de gewrichten	O	O	O	O	O	O
22. Spierpijn (niet door sporten)	O	O	O	O	O	O
23. Hartkloppingen	O	O	O	O	O	O
24. Misselijkheid	O	O	O	O	O	O
25. Opkomend maagzuur	O	O	O	O	O	O
26. Verminderde eetlust	O	O	O	O	O	O
27. Overgeven/braken	O	O	O	O	O	O

E) VRAGEN OVER LUCHTWEGKLACHTEN VAN FAMILIE

E1) Heeft uw vader of moeder ooit astma gehad?

- o Ja
- o Nee
- o Weet ik niet

E2) Heeft uw vader of moeder ooit ‘hooikoorts’ of een andere neusallergie gehad?

- o Ja
- o Nee
- o Weet ik niet

E3) Hoeveel broers of zussen heeft u of heeft u gehad?

- o Geen → Ga door naar vraag E4
- o .... (aantal broers en zussen) → Ga door naar vraag E3 a

E3 a) Hebben uw broers of zussen ooit astma gehad?

- o Ja
- o Nee
- o Weet ik niet

E3 b) Hebben uw broers of zussen ooit ‘hooikoorts’ of een andere neusallergie gehad?

- o Ja
- o Nee
- o Weet ik niet

E4) Zijn er, bij uw weten, ooit familieleden (grootouders, ouders, broers of zussen) gestopt met werken op een boerderij/agrarisch bedrijf vanwege luchtwegklachten of allergie?

- o Ja
- o Nee
- o Weet ik niet
- o Mijn familieleden hebben nooit op een boerderij/agrarisch bedrijf gewerkt

F) VRAGEN OVER ROKEN

F1) Heeft u gedurende uw hele leven meer dan 100 sigaretten/50 sigaren/500 gram pijptabak gerookt?

- o Ja, ik heb vroeger gerookt, maar ik ben nu gestopt met roken
- o Ja, ik rook nog steeds
- o Nee

F2) Wilt u aangeven hoeveel sigaretten, sigaren en gram pijp tabak u gemiddeld per dag rookt (of rookte, toen u rookte)? (1 pakje shag = 40 sigaretten)

- o Sigaretten .... per dag
- o Sigaren .... per dag
- o Pijp tabak .... gram per dag

F2 a) Hoe oud was u toen u begon te roken? .... jaar

F2 c) Indien van toepassing, hoe oud was u toen u (voor de laatste keer) stopte met roken?
.... jaar

Heeft u nog iets toe te voegen aan deze vragenlijst met betrekking tot u werkomgeving of uw gezondheid of heeft u nog opmerkingen of suggesties met betrekking tot deze vragenlijst?

……………………………………………………………………………………………………

……………………………………………………………………………………………………
