# Supplementary material for: Occupational endotoxin exposure in association with atopic sensitization and respiratory health in adults: Results of a 5-year follow-up
Source: PLoS One. 2017 Dec 6;12(12):e0189097. doi: 10.1371/journal.pone.0189097 (PMC5718503; doi:10.1371/journal.pone.0189097)
Supplement: S2 Text — (PDF) [file pone.0189097.s002.pdf]

Questionnaire on work and health for farmers and agricultural industry workers

In this questionnaire you will find questions about your personal information and your work activities (part A, B and C), questions about respiratory health and allergies (part D and E), and about your smoking habits (part F).
Parts A, D, E and F are the same for all participants, parts B and C differ per sector.

If anything is unclear, you can always call us or send an email.

Institute for Risk Assessment Sciences
Utrecht University

Universiteit Utrecht

In case you do not want to fill in this questionnaire, we ask you to return the – uncompleted - questionnaire, including the reason you don't want to fill it in. This way, we will know whether all questionnaires have reached their destination and we can estimate whether the reasons people do not participate are of influence on the conclusions of this study.

- I do not want to complete this questionnaire, because ....……

  ....

All participants

A) General Questions

A1) What is your gender?
- o Male
- o Female

A2) What is your date of birth? _____ _____ 19______ (day, month, year)

A3) Did you grow up on a farm/agricultural company?
- o Yes→ Continue to question A3 a
- o No → Continue to question B1

A3 a) What was the main production activity in the farm/company that you grew up in? *(Multiple answers possible)*
- o Livestock
- o Agriculture

Farmers

B) Questions about your company

(Part B only needs to be completed by one of the owners of the farm)

B1) How many people work on the farm including yourself? ………… (number)

B2) Was you farm previously a regular farm and did you then switch to organic farming?

- Yes → Continue to question B2a
- No → Continue to question B3

B2a) How many years ago did you start changing to organic production? ………. (years ago)

B3) What is or what are the main production activities in your current company? Are these activities organic, biodynamic, partly organic or regular in nature? *(Multiple answers possible)*

	Type of production	Organic	Biodynamic	Partly organic	Regular
O	Dairy farm	O	O	O	O
O	Cattle farm, meat production	O	O	O	O
O	Poultry farm, laying hens	O	O	O	O
O	Poultry farm, broilers	O	O	O	O
O	Breeding sows	O	O	O	O
O	Fattening pigs	O	O	O	O
O	Sheep or goat farm, dairy	O	O	O	O
O	Sheep or goat farm, meat	O	O	O	O
O	Agriculture: grain	O	O	O	O
O	Agriculture: fodder crops	O	O	O	O
O	Agriculture: other	O	O	O	O
O	Open air vegetable growth	O	O	O	O
O	Flower bulbs	O	O	O	O
O	Trees	O	O	O	O
O	Fruit	O	O	O	O
O	Horticulture	O	O	O	O
O	Mushrooms	O	O	O	O
O	Other, i.e.	O	O	O	O

B4) What number of the below listed farm animals do you keep on your farm?

Farm animal	Number
Cows	....
Bulls	....
Calf and heifers	....
Laying hen	....
Broilers	....
Other poultry, i.e.	....
Breeding sow	....
Fattening pigs	....
Goats	....
Sheep	....
Horses	....
Other animals, i.e.	....
Other animals, i.e.	....

B5) Do you use disinfectants at your farm?
- Yes → Continue to question B5 a
- No → Continue to question C1

B5 a) Which disinfectants do you use?
Only tick the boxes of the disinfectants you use regularly
(Multiple answers possible)

O	QAC (Deosan, P3-Incidin)
O	QAC + bases (Incosept, Nofordes)
O	QAC + aldehydes (Macrodes, Megades, Favoriet Ontsmetal, Parvotech, Aldekol, CID 20, Neoseptal, Roloxid, Togodor)
O	Chloramine-T (Halamid)
O	Chlorine containing compounds (Actisan, Sumatox, Chloortabletten, natriumhypochloriet)
O	Bases+chlorine containing compounds (Quick Clean, Neomosan)
O	Other disinfectants, i.e. …………………………………………………………………..

Farmers

C) Questions about your work

C1) At what age did you start working at an agricultural company? ……….. (years)

C2) At what age did you start working at the current agricultural company? ……….. (years)

C3) Are you (co)owner of the company where you currently work?

- o Yes
- o No

C4) How many hours a week do you work at your current company?

- o Less than 20 hours a week
- o 20 to 32 hours a week
- o 33 to 40 hours a week
- o More than 40 hours a week

C5) Which of the below tasks **do you perform yourself**?
(Multiple answers possible)

Task			
O	Animal care	O	Working with liquid manure
O	Working with wood chips or sawdust	O	Working with dry manure
O	Working with straw	O	Working with pesticides
O	Working with hay	O	Taking care of crops, outside
O	Working with silage grass	O	Taking care of crops, horticulture
O	Personally mixing animal fodder	O	Working with grain
O	Desinfecting areas	O	Sorting onions or flower bulbs
O	Cleaning with high-pressure cleaner	O	Sorting potatoes
O	Cleaning with fire hose	O	Other tasks at agricultural farm
O	None of the above, my main task at the company is: …………………………………..………………………………		

C6) What is the most dusty task you perform, according to you?

…………………………………………..…………………….…………………….…………………

C7) Do you regularly use personal protective equipment during your work?
(Multiple answers possible)

- o Yes, gloves
- o Yes, respiratory protection
- o No

Flower bulb industry

B) Questions about your work

B1) When did you start working at the current company?

_____ _____ ______ (day, month, year)

B2) How many hours a week do you work?

- o Less than 20 hours a week
- o 20 to 32 hours a week
- o 33 to 40 hours a week
- o More than 40 hours a week

B3) What is your current job title?

o Forklift driver
o Production worker
o Packaging worker
o Technical service, maintenance
o Housekeeping
o Cultivation-/harvest worker
o Lab technician
o Administrative employee
o Supervisor (Company director, Managing director)
o Other, i.e.:……………………………

B4) Which of the following tasks do you perform and how often?

	Never	At most half a day per week	1 to 3 days a week	4 to 5 days a week
○ Taking care of crops, culture, harvest	○	○	○	○
○ Fork lift driving	○	○	○	○
○ Rinsing flower bulbs	○	○	○	○
○ Sorting flower bulbs	○	○	○	○
○ Packaging, by hand	○	○	○	○
○ Packaging, automated	○	○	○	○
○ Repair, maintenance	○	○	○	○
○ Cleaning: cleaning up/sweeping	○	○	○	○
○ Cleaning: high pressure spray	○	○	○	○
○ Cleaning: compressed air	○	○	○	○
○ Cleaning: fire hose	○	○	○	○
○ Laboratory activities	○	○	○	○
○ Office work	○	○	○	○
○ Other, i.e..	○	○	○	○

B5) Where in the company do you work most of the time (<u>tick only one box</u>)?

○ Outside
○ Reception hall
○ Production department
○ Packaging department
○ Logistics department/warehouse
○ Workshop
○ Laboratory
○ Office
○ Somewhere else i.e.:…………………………..

Animal feed

B) Questions about your work

B1) When did you start working at the current company?

_____ _____ ______ (day, month, year)

B2) How many hours a week do you work?

- ○ Less than 20 hours a week
- ○ 20 to 32 hours a week
- ○ 33 to 40 hours a week
- ○ More than 40 hours a week

B3) What is your current job title?

○ Truck driver
○ Crane driver
○ Ship unloader (bobcat)
○ Production worker / process operator
○ Filling bags
○ Forklift driver
○ Technical service, maintenance
○ Silo cleeaner
○ Lab / Quality worker
○ Administrative assistant
○ Supervisor (Company director, Managing director)

B4) Which of the following tasks do you perform and how often?

	Never	At most half a day per week	1 to 3 days a week	4 to 5 days a week
○ Unloading raw materials	○	○	○	○
○ Control room	○	○	○	○
○ Weighing and adding special ingredients	○	○	○	○
○ Forklift driving	○	○	○	○
○ Loading and filling-up bags	○	○	○	○
○ Truck loading	○	○	○	○
○ Repair, maintenance	○	○	○	○
○ Cleaning: cleaning up/sweeping	○	○	○	○
○ Cleaning: vacuuming	○	○	○	○
○ Cleaning: compressed air (clothing)	○	○	○	○
○ Laboratory work	○	○	○	○
○ Officework	○	○	○	○
○ Machinery pressing, unloading, mixing, sacking	○	○	○	○

B5) Where in the company do you work most of the time (tick only one box)?

○ Outside (unloading ships)
○ (Un)loading bulk transport trucks
○ Control room
○ Factory floor
○ Sacking department
○ Dispatch room
○ Workshop
○ Laboratory
○ Office

Union industry

B) Questions about your work

B1) When did you start working at the current company?

_____ _____ ______ (day, month, year)

B2) How many hours a week do you work?

- o Less than 20 hours a week
- o 20 to 32 hours a week
- o 33 to 40 hours a week
- o More than 40 hours a week

B3) What is your current job title?

o Forklift driver
o Production worker
o Packaging
o Technical service, maintenance
o Housekeeping
o Cultivation-/harvest worker
o Lab technician
o Administrative employee
o Supervisor (Company director, Managing director)
o Other, i.e.:……………………………

B4) Which of the following tasks do you perform and how often?

	Never	At most half a day per week	1 to 3 days a week	4 to 5 days a week
○ Taking care of crops, cultivation, harvesting	○	○	○	○
○ Forklift driving	○	○	○	○
○ Onion cleaning	○	○	○	○
○ Onion sorting	○	○	○	○
○ Packing	○	○	○	○
○ Repair, maintenance	○	○	○	○
○ Cleaning: cleaning up/sweeping	○	○	○	○
○ Cleaning: high pressure spray	○	○	○	○
○ Cleaning: compressed air	○	○	○	○
○ Cleaning: fire hose	○	○	○	○
○ Laboratory activities	○	○	○	○
○ Office work	○	○	○	○
○ Other, i.e..	○	○	○	○

B5) Where in the company do you work most of the time (<u>tick only one box</u>)?

○ Outside
○ Crop reception
○ Production
○ Packaging
○ Logistics department/warehouse
○ Workshop
○ Laboratory
○ Office
○ Somewhere else i.e.:…………………………..

Seed industry

B) Questions about your work

B1) When did you start working at the current company?

______ ______ _______ (day, month, year)

B2) How many hours a week do you work?

- Less than 20 hours a week
- 20 to 32 hours a week
- 33 to 40 hours a week
- More than 40 hours a week

B3) What is your current job title?

○ Forklift driver
○ Production worker/ process operator
○ Packaging
○ Technical service, maintenance
○ Housekeeping
○ Cultivation worker
○ Lab technician
○ Administrative employee
○ Supervisor (Company director, Managing director)
○ Other, i.e.:……………………………

B4) Which of the following tasks do you perform and how often?

	Never	At most half a day per week	1 to 3 days a week	4 to 5 days a week
○ Taking care of crops, cultivation, harvesting	○	○	○	○
○ Forklift driving	○	○	○	○
○ Tilting / decanting	○	○	○	○
○ Cleaning	○	○	○	○
○ Mixing	○	○	○	○
○ Seed disinfecting, coating	○	○	○	○
○ Packaging	○	○	○	○
○ Repair, maintenance	○	○	○	○
○ Cleaning: cleaning up/sweeping	○	○	○	○
○ Cleaning: vacuuming	○	○	○	○
○ Cleaning: compressed air	○	○	○	○
○ Laboratory activities	○	○	○	○
○ Office work	○	○	○	○
○ Other, i.e..	○	○	○	○

B5) Where in the company do you work most of the time (<u>tick only one box</u>)?

○ Outside
○ Greenhouse
○ Crop reception
○ Production floor
○ Packaging department
○ Logistics department/warehouse
○ Workshop
○ Laboratory
○ Office
○ Somewhere else i.e.:…………………………..

All company workers (not farmers)

B6) What is the most dusty work you perform, according to you?
………………………………..………..………………………..…………………….…………………

B7) Do you regularly use personal protective equipment during your work?
(<u>Multiple answers possible</u>)

- o Yes, gloves
- o Yes, respiratory protection
- o No

B8) Before you started this job, did you work at another company in the flowerbulb/animal feed/ onion/seed industry?

- o Yes → How long? …………Years
- o No

B9) Before you started work in the flowerbulb/animal feed/ onion/seed industry, did you have another job <u>at which you were exposed to dust?</u>
E.g. bakery, grain industry, agriculture, etc.

- o Yes → Continue to question B9a
- o No

B9 a) What company or companies?

…………………………………………..	How long?	…………Years
…………………………………………..	How long?	…………Years
…………………………………………..	How long?	…………Years

All company workers (not farmers)

C) Questions about personal hygiene

C1) Do you ever eat or drink at your work station (so outside the office or canteen)?

- Yes, always
- Yes, some times
- No

C2) Do you usual shower at the end of your work day?

- Yes, at work
- Yes, at home
- No

C3) Does your company wash your work clothes or do you wash them at home?

- The company
- Partly by the company, partly at home
- At home

C4) Do you change clothes at home or at work?

- At work
- At home

All participants

D) Questions about health

D1) Do you ever have problems breathing?

- Continuously, my breath is never completely fine
- Repeatedly, but it always recovers completely
- Never/rarely

D2) In the past 12 months, did you cough almost daily for three consecutive months?

- Yes
- No

D3) In the past 12 months, did you ever wake up due to coughing?

- Yes
- No

D4) In the past 12 months, did you cough up phlegm almost daily for 3 consecutive months?

- Yes
- No

D5) In the past months, did you ever wake up with chest tightness?

- Yes
- No

D6) Are you troubled by shortness of breath when hurrying on level ground, or when walking up a slight incline or taking the stairs at a normal pace?

- Yes → Continue to question D6 a
- No → Continue to question D7

D6a) Do you get short of breath walking with other people of your own age on level ground in normal pace?

- Yes
- No

D7) Have you had attacks of wheezing at any time in the last 12 months?

- Yes → Continue to question D7 a
- No → Continue to question D8

D7 a) Were you troubled with shortness of breath during this wheezing?

- Yes
- No

D7 b) Have you had this wheezing without having a cold?

- Yes
- No

D8) Did you ever have to change jobs due to bronchitis or emphysema?

- o Yes
- o No

D9) In the last 12 months, did you use medicines for bronchitis or emphysema?

- o Yes → Continue to question D9 a
- o No → Continue to question D10

D9 a) What medicines have you used for bronchitis or emphysema?
Multiple answers possible

- o Ventolin
- o Atrovent
- o Bricanyl
- o Prednison
- o Other, i.e. ....

D10) Have you at any time in the last 12 months been woken at night by a attack of shortness of breath?

- o Yes
- o No

D11) Were you at any time in the last 12 months troubled by an attack of shortness of breath during the day while at rest?

- o Yes
- o No

D12) Were you at any time in the last 12 months troubled by an attack of shortness of breath after exercise?

- o Yes
- o No

D13) Have you ever had asthma?

- o Yes → Continue to question D13 a
- o No → Continue to question D14

D13 a) Was the asthma confirmed by a doctor?

- o Yes → Continue to question D13 b
- o No → Continue to question D13 c

D13 b) How old were you when diagnosed with asthma? …………….year

D13 c) Have you had an asthma attack in the last 12 months?

- o Yes → Continue to question D13 d
- o No → Continue to question D13 f

D13 d) How many asthma attacks have you had in the past 12 months? …………….

D13 e) Have you had an asthma attack during work in the past 12 months?
- o Yes
- o No

D13 f) Are you currently using medicines for asthma (inhalers, aerosols, tablets)?
- o Yes → Continue to question D13 g
- o No → Continue to question D14

D13 g) What medicines have you used for asthma?
Multiple answers possible
- o Ventolin
- o Pulmicort
- o Atrovent
- o Bricanyl
- o Becotide
- o Symbicort
- o Flixotide
- o Prednison
- o Other, i.e. ....

D14) Are you or have you been hypersensitive or allergic to one or more substances?
- o Yes → Continue to question D14 a
- o No → Continue to question D15

D14 a) If yes, please tick the applicable boxes below and indicate which symptoms you have?
Multiple answers possible.

		Sneezing or runny nose	Breathlessness (asthma)	Itchy or red skin	Itchy or teary eyes
O	House dust	O	O	O	O
O	Food	O	O	O	O
O	Animals	O	O	O	O
O	Plants or grass pollen	O	O	O	O

D14 b) In the last 12 months, have you been to a general physician or specialist due to your allergic symptoms?
- o Yes
- o No

D14 c) In the last 12 months, have you ever had to leave your work due to allergic symptoms?
- o Yes
- o No

D15) Have you ever had to change jobs because of asthma?
- o Yes
- o No

D16) Have you ever had to change jobs due to allergy?

- o Yes
- o No

D17) Have you ever had to change jobs due to other health problems?

- o Yes
- o No

D18) Have you ever had wheeze, shortness of breath or chest tightness due to certain work tasks or workplaces in the past 12 months?

- o Yes → Continue to question D18 a
- o No → Continue to question D19

D18 a) What workplaces or work tasks do you associate with these symptoms?

………………………………………………………………………………………

D18 b) When did the symptoms begin?

- o Within 4 hours of starting work
- o More than 4 hours after starting work

D19) In the last 12 months, have you experienced sudden onset of flu-like symptoms like fever, shivering, malaise, muscle or joint ache, <u>from which you recovered completely within 1-2 days?</u>

- o Yes → Continue to question D19 a
- o No → Continue to question D20

D19 a) What workplaces or work tasks do you associate with these symptoms?

………………………………………………………………………………………

D19 b) How often did this happen in the last 12 months? …………… times

D 20) In the last 12 months, how often have you suffered from one or more of below symptoms?
AND
Do these symptoms get worse during or after your work?

	How often did you have this symptom?				Does it get worse during or after work?	
	Daily or almost daily	1 to 2 times a week	1 to 2 times a month	(almost) never	No	Yes
1. Dry cough	O	O	O	O	O	O
2. Coughing up phlegm	O	O	O	O	O	O
3. Wheezing	O	O	O	O	O	O
4. Shortness of breath with wheezing	O	O	O	O	O	O
5. Shortness of breath	O	O	O	O	O	O
6. Chest tightness	O	O	O	O	O	O
7. Stuffy nose	O	O	O	O	O	O
8. Runny nose	O	O	O	O	O	O
9. Itchy nose or sneezing	O	O	O	O	O	O
10. Dry throat or throat ache	O	O	O	O	O	O
11. Itchy, watering eyes	O	O	O	O	O	O
12. Itchy or red skin for more than 2 days	O	O	O	O	O	O
13. Skin rash	O	O	O	O	O	O
14. Headache	O	O	O	O	O	O
15. Heavy/oppressive feeling in your head	O	O	O	O	O	O
16. Dizziness	O	O	O	O	O	O
17. Unusual fatigue	O	O	O	O	O	O
18. Difficulty concentrating	O	O	O	O	O	O
19. Fever	O	O	O	O	O	O
20. Chills, attacks of shivering	O	O	O	O	O	O
21. Joint pain	O	O	O	O	O	O
22. Muscle pain (not sport related)	O	O	O	O	O	O
23. Palpitation of the heart	O	O	O	O	O	O
24. Nausea	O	O	O	O	O	O
25. Heartburn	O	O	O	O	O	O
26. Loss of appetite	O	O	O	O	O	O
27. Vomiting	O	O	O	O	O	O

All participants

E) Questions about respiratory problems in your family

E1) Has your father or mother ever had asthma?

- Yes
- No
- I don't know

E2) Has your father or mother ever had 'hay fever' or another nose allergy?

- Yes
- No
- I don't know

E3) How many siblings do you have or have you had?

- None → Continue to question E4
- .... (number of siblings) → Continue to question E3 a

E3 a) Have your brothers or sisters ever had asthma?

- Yes
- No
- I don't know

E3 b) Have your brothers or sisters ever had 'hay fever' or another nose allergy?

- Yes
- No
- I don't know

E4) As far as you know, have one of your family members (grandparents, parents or siblings) ever stopped working on a farm / agricultural company due to respiratory symptoms or allergy?

- Yes
- No
- I don't know
- My family members have never worked on a farm/agricultural company

All participants

F) Questions about smoking

F1) During your entire life, have you smoked more than 100 cigarettes/ 50 cigars /500 grams of pipe tobacco?

- o Yes, I used to smoke, but I have now stopped smoking
- o Yes, I still smoke
- o No

F2) Can you indicate how many cigarettes, cigars and grams of pipe tobacco you smoke on a daily basis (or smoked when you used to smoke)? (1 pack of shag = 40 cigarettes)

- o cigarettes .... per day
- o cigars .... per day
- o Pipe tobacco .... grams per day

F2 a) How old were you when you started smoking? .... years

F2 c) If applicable: how old were you when you (for the last time) quit smoking?
.... years

Do you have anything to add to this questionnaire with regard to your work environment or your health, or do you have any remarks or suggestions with regard to this questionnaire?

………

………
