# Supplementary material for: Occupational endotoxin exposure in association with atopic sensitization and respiratory health in adults: Results of a 5-year follow-up
Source: PLoS One. 2017 Dec 6;12(12):e0189097. doi: 10.1371/journal.pone.0189097 (PMC5718503; doi:10.1371/journal.pone.0189097)
Supplement: S1 Table — (PDF) [file pone.0189097.s003.pdf]

Exposure estimates using baseline model and pooled model by industry and job title

Baseline exposure model is based on baseline measurements only, pooled model is based on all measurements from both time points. The pooled model is used to assign exposure to participants in the current study population.

		Baseline model		Pooled model	
**Sector**	**Jobtitle**	**n**	**Endotoxin [EU.m^{-3}]**	**n**	**Endotoxin [EU.m^{-3}]**
**Flower bulb**					
	Office	3	14	8	13
	Lifttruck	2	86	3	206
	Technician	2	93	3	103
	Packer	9	169	9	182
	Crop grower	1	181	2	581
	Operator	14	720	28	443
**Animal feed**					
	Office	6	35	8	32
	Lab	4	59	4	59
	Truckdriver	10	251	10	251
	Controlroom	19	289	28	220
	Operator	27	335	33	343
	Technician	7	416	9	407
	Cleaner	3	946	3	966
	Crane driver	5	1086	5	1045
	Unloader ships	5	10645	5	10305
	Lifttruck			2	674
	Packer			2	1444
**Onions (modernized)**					
	Technician			1	9006
	Office	3	381	7	336
	Packer	1	754	1	625
	Lifttruck	2	775	6	846
	Operator	6	1453	8	1747
**Onions (traditional)**					
	Technician	2	801	3	1175
	Packer	4	4906	7	6474
	Lifttruck	6	5045	9	4131
	Operator	15	9454	26	7764
**Seeds**					
	Lab	2	22	6	23
	Packer	2	25	2	20
	Crop grower	2	36	4	35
	Operator	5	406	12	279
**Farmer**					
	Crop	30	63	40	74
	Livestock	46	219	71	246
	Swine	6	3395	11	2876
