# Supplementary material for: Occupational endotoxin exposure in association with atopic sensitization and respiratory health in adults: Results of a 5-year follow-up
Source: PLoS One. 2017 Dec 6;12(12):e0189097. doi: 10.1371/journal.pone.0189097 (PMC5718503; doi:10.1371/journal.pone.0189097)
Supplement: S2 Table — (PDF) [file pone.0189097.s004.pdf]

Adjusted logistic regression analysis of reported health symptoms and specific IgE positivity in association with endotoxin exposure with and without inclusion of Δexposure. OR's are associated with a 2-fold increase in endotoxin exposure. Analyses are adjusted for potential confounders: age, gender and smoking, and wheeze is additionally adjusted for atopy.

			Baseline exposure		Baseline exposure and Δexposure			
					Baseline exposure		Δexposure	
		N	OR	95% CI	OR	95% CI	OR	95% CI
**Asthma**	n-n	210						
	n-y	4	n<5 data not shown					
	y-y	8						
	y-n	12	0.74	(0.46 - 1.20)	0.82	(0.47 - 1.43)	1.15	(0.77 - 1.71)
	n-n	210						
	y-y	8	1.11	(0.83 - 1.49)	1.11	(0.83 - 1.49)	1.00	(0.86 - 1.18)
**Wheeze**	n-n	168						
	n-y	18	0.88	(0.70 - 1.11)	0.87	(0.69 – 1.09)	0.93	(0.81 - 1.08)
	y-y	19						
	y-n	7	0.74	(0.42 - 1.29)	*0.22*	*(0.04 – 1.22)*	*0.53*	*(0.25 - 1.10)*
	n-n	168						
	y-y	19	1.13	(0.91 - 1.40)	1.13	(0.91–1.4)	1.01	(0.89 - 1.13)
**Allergy**	n-n	153						
	n-y	16	1.07	(0.84 - 1.35)	1.08	(0.85 - 1.37)	1.09	(0.98 - 1.22)
	y-y	52						
	y-n	13	1.03	(0.78 - 1.35)	0.99	(0.74 - 1.32)	0.91	(0.78 - 1.07)
	n-n	153						
	y-y	52	0.92	(0.80 - 1.06)	0.93	(0.81 - 1.07)	1.03	(0.95 - 1.11)
**Hay fever**	n-n	197						
	n-y	9	0.87	(0.65 - 1.17)	0.87	(0.65 - 1.17)	1.00	(0.87 - 1.16)
	y-y	19						
	y-n	9	*2.19*	*(0.96 - 4.99)*	1.85	(0.81 - 4.22)	0.74	(0.49 - 1.10)
	n-n	197						
	y-y	19	**0.68**	**(0.54 - 0.87)**	**0.69**	**(0.54 - 0.87)**	1.01	(0.89 - 1.15)
**Atopy**	n-n	136						
	n-y	10	*0.75*	*(0.55 - 1.02)*	*0.72*	*(0.52 - 1.00)*	0.93	(0.79 - 1.10)
	y-y	57						
	y-n	9	1.03	(0.73 - 1.46)	1.02	(0.71 - 1.45)	1.07	(0.87 - 1.31)
	n-n	136						
	y-y	57	0.89	(0.77 - 1.03)	0.90	(0.77 - 1.04)	1.05	(0.97 - 1.14)
**HDM IgE**	n-n	162						
	n-y	3	n<5 data not shown					
	y-y	40						
	y-n	7	0.91	(0.62 - 1.34)	0.90	(0.61 - 1.34)	0.94	(0.76 - 1.17)
	n-n	162						
	y-y	40	1.05	(0.90 - 1.22)	1.06	(0.91 - 1.24)	*1.08*	*(0.99 - 1.17)*
**Grass IgE**	n-n	162						
	n-y	9	0.83	(0.61 - 1.12)	0.82	(0.60 - 1.12)	1.13	(0.96 - 1.33)
	y-y	32						
	y-n	9	1.24	(0.76 - 2.02)	1.18	(0.72 - 1.94)	1.11	(0.88 - 1.41)
	n-n	162						
	y-y	32	**0.81**	**(0.68 - 0.97)**	**0.82**	**(0.69 - 0.98)**	1.06	(0.96 - 1.17)
