# Supplementary material for: Occupational endotoxin exposure in association with atopic sensitization and respiratory health in adults: Results of a 5-year follow-up
Source: PLoS One. 2017 Dec 6;12(12):e0189097. doi: 10.1371/journal.pone.0189097 (PMC5718503; doi:10.1371/journal.pone.0189097)
Supplement: S3 Table — (PDF) [file pone.0189097.s005.pdf]

Linear regression analysis of lung function and total IgE in association with endotoxin exposure with and without inclusion of Δexposure. Betas are associated with a 2-fold increase in endotoxin exposure. Analyses are all adjusted for possible confounders (age, gender and smoking).

		**Baseline exposure**		**Baseline exposure and Δ exposure**			
				**Baseline exposure**		**Δ exposure**	
	**N**	**beta**	**p**	**beta**	**p**	**beta**	**p**
Δ FEV1							
mL	161	3.50	0.18	2.87	0.28	-1.88	0.17
% pred (GLI)	161	0.03	0.70	0.01	0.83	-0.03	0.41
Δ FVC							
mL	161	0.71	0.84	-0.54	0.88	-3.86	**0.03**
% pred (GLI)	161	-0.04	0.55	-0.06	0.38	-0.06	*0.10*
ΔFEV1/FVC							
x100	161	0.05	*0.07*	0.06	*0.05*	0.02	0.14
% pred (GLI)	161	0.07	*0.06*	0.08	**0.04**	0.03	0.13
ΔFEF25-75							
	161	0.01	**0.01**	0.01	**0.01**	0.00	0.86
% pred (GLI)	161	0.28	**0.03**	0.27	**0.04**	0.01	0.85
Δ Total IgE	212	0.03	0.80	0.03	0.68	0.04	0.40
