# Supplementary material for: Occupational endotoxin exposure in association with atopic sensitization and respiratory health in adults: Results of a 5-year follow-up
Source: PLoS One. 2017 Dec 6;12(12):e0189097. doi: 10.1371/journal.pone.0189097 (PMC5718503; doi:10.1371/journal.pone.0189097)
Supplement: S4 Table — (PDF) [file pone.0189097.s006.pdf]

Adjusted logistic regression analysis of reported health symptoms and specific IgE positivity in association with endotoxin exposure with and without additional adjustment for farm childhood. OR's are associated with a 2-fold increase in endotoxin exposure. Analyses are adjusted for potential confounders: age, gender and smoking, and wheeze is additionally adjusted for atopy.

		New onset vs never				Loss vs persistent				Persistent vs never			
		Not adjusted for farm childhood		Adjusted for farm childhood		Not adjusted for farm childhood		Adjusted for farm childhood		Not adjusted for farm childhood		Adjusted for farm childhood	
		OR	95% CI	OR	95% CI	OR	95% CI	OR	95% CI	OR	95% CI	OR	95% CI
**Self-reported symptoms**													
Asthma	baseline exposure	*n < 5*		*n < 5*		0.73	(0.46 - 1.15)	0.74	(0.46 - 1.2)	1.12	(0.82 - 1.52)	1.11	(0.83 - 1.49)
	farm childhood	*data not shown*		*data not shown*		-		1.62	(0.15 - 17.78)	-		0.27	(0.05 - 1.44)
Wheeze	baseline exposure	0.88	(0.70 - 1.11)	0.88	(0.70 - 1.11)	0.78	(0.48 - 1.26)	0.74	(0.42 - 1.29)	1.12	(0.90 - 1.40)	1.13	(0.91 - 1.40)
	farm childhood	-		1.27	(0.41 - 3.95)	-		3.77	(0.38 - 37.22)	-		0.61	(0.21 - 1.81)
Allergy	baseline exposure	1.07	(0.84 - 1.36)	1.07	(0.84 - 1.35)	1.03	(0.79 - 1.34)	1.03	(0.78 - 1.35)	0.93	(0.80 - 1.07)	0.92	(0.80 - 1.06)
	farm childhood	-		0.68	(0.23 - 1.99)	-		2.55	(0.66 - 9.92)	-		0.56	(0.28 - 1.09)
Hay fever	baseline exposure	0.87	(0.64 - 1.19)	0.87	(0.65 - 1.17)	1.83	(0.97 - 3.44)	2.19	(0.96 - 4.99)	**0.67**	**(0.53 - 0.85)**	**0.68**	**(0.54 - 0.87)**
	farm childhood	-		0.30	(0.07 - 1.34)	-		4.45	(0.44 - 45.09)	-		0.54	(0.19 - 1.51)
**Sensitization**													
Grass IgE	baseline exposure	0.84	(0.60 - 1.16)	0.83	(0.61 - 1.12)	1.40	(0.89 - 2.21)	1.24	(0.76 - 2.02)	**0.81**	**(0.68 - 0.97)**	**0.81**	**(0.68 - 0.97)**
	farm childhood	-		0.31	(0.08 - 1.30)	-		7.43	(0.67 - 81.7)	-		**0.27**	**(0.11 - 0.64)**
HDM IgE	baseline exposure	*n < 5*		*n < 5*		0.91	(0.63 - 1.33)	0.91	(0.62 - 1.34)	1.05	(0.90 - 1.22)	1.05	(0.90 - 1.22)
	farm childhood	*data not shown*		*data not shown*		-		1.49	(0.26 - 8.61)	-		0.56	(0.27 - 1.17)
Atopy*	baseline exposure	0.75	(0.55 - 1.04)	0.75	(0.55 - 1.02)	1.08	(0.80 - 1.47)	1.03	(0.73 - 1.46)	0.89	(0.77 - 1.03)	0.89	(0.77 - 1.03)
	farm childhood	-		**0.24**	**(0.06 - 0.97)**	-		8.40	(0.92 - 76.91)	-		**0.34**	**(0.17 - 0.68)**

*Positive for any of the four allergens (cat, dog, grass or HDM).
