# Supplementary material for: Occupational endotoxin exposure in association with atopic sensitization and respiratory health in adults: Results of a 5-year follow-up
Source: PLoS One. 2017 Dec 6;12(12):e0189097. doi: 10.1371/journal.pone.0189097 (PMC5718503; doi:10.1371/journal.pone.0189097)
Supplement: S5 Table — (PDF) [file pone.0189097.s007.pdf]

Linear regression analysis of lung function and total IgE in association with endotoxin exposure with and without additional adjustment for farm childhood: association of change in lung function or total IgE with endotoxin exposure. Betas are associated with a 2-fold increase in endotoxin exposure. Analyses are all adjusted for possible confounders (age, gender and smoking).

		Not adjusted for farm childhood		Adjusted for farm childhood	
		beta	p	beta	p
**Δ FEV1**					
mL .year^{-1}	baseline exposure	3.35	0.20	3.38	0.20
	farm childhood	-		19.54	0.08
% pred (GLI) .year^{-1}	baseline exposure	0.02	0.75	0.02	0.74
	farm childhood	-		0.37	0.20
**Δ FVC**					
mL .year^{-1}	baseline exposure	0.45	0.90	0.51	0.89
	farm childhood	-		42.83	**0.01**
% pred (GLI) .year^{-1}	baseline exposure	-0.05	0.51	-0.05	0.52
	farm childhood	-		0.71	**0.02**
**ΔFEV1/FVC**					
.year^{-1}	baseline exposure	$0.05 \cdot 10^{-3}$	0.07	$0.05 \cdot 10^{-3}$	0.07
	farm childhood	-		-0.19	0.13
% pred (GLI) .year^{-1}	baseline exposure	0.07	0.06	0.07	0.06
	farm childhood	-		-0.23	0.13
**ΔFEF25-75**					
.year^{-1}	baseline exposure	0.01	**0.01**	0.01	**0.01**
	farm childhood	-		0.00	0.98
% pred (GLI) .year^{-1}	baseline exposure	0.27	**0.04**	0.27	**0.04**
	farm childhood	-		-0.13	0.82
**Δ Total IgE**					
IU .year^{-1}	baseline exposure	-0.01	0.87	-0.01	0.90
	farm childhood	-		-0.85	**0.02**
